# A multidisciplinary systematic review of the use of diagrams as a means of collecting data from research subjects: application, benefits and recommendations

**DOI:** 10.1186/1471-2288-11-11

**Published:** 2011-01-27

**Authors:** Muriah J Umoquit, Peggy Tso, Helen ED Burchett, Mark J Dobrow

**Affiliations:** 1 Cancer Services & Policy Research Unit, Cancer Care Ontario, (620 University Ave), Toronto, (M5G 2L7), Canada; 2 Department of Health Policy, Management and Evaluation, University of Toronto, (155 College Street), Toronto, (M5T 3M6), Canada; 3 Department of Global Health and Development, Faculty of Public Health and Policy, London School of Hygiene and Tropical Medicine, (15-17 Tavistock Place), London, (WC1H 9SH), UK

## Abstract

**Background:**

In research, diagrams are most commonly used in the analysis of data and visual presentation of results. However there has been a substantial growth in the use of diagrams in earlier stages of the research process to collect data. Despite this growth, guidance on this technique is often isolated within disciplines.

**Methods:**

A multidisciplinary systematic review was performed, which included 13 traditional healthcare and non-health-focused indexes, non-indexed searches and contacting experts in the field. English-language articles that used diagrams as a data collection tool and reflected on the process were included in the review, with no restriction on publication date.

**Results:**

The search identified 2690 documents, of which 80 were included in the final analysis. The choice to use diagrams for data collection is often determined by requirements of the research topic, such as the need to understand research subjects' knowledge or cognitive structure, to overcome cultural and linguistic differences, or to understand highly complex subject matter. How diagrams were used for data collection varied by the degrees of instruction for, and freedom in, diagram creation, the number of diagrams created or edited and the use of diagrams in conjunction with other data collection methods. Depending on how data collection is structured, a variety of options for qualitative and quantitative analysis are available to the researcher. The review identified a number of benefits to using diagrams in data collection, including the ease with which the method can be adapted to complement other data collection methods and its ability to focus discussion. However it is clear that the benefits and challenges of diagramming depend on the nature of its application and the type of diagrams used.

**Discussion/Conclusion:**

The results of this multidisciplinary systematic review examine the application of diagrams in data collection and the methods for analyzing the unique datasets elicited. Three recommendations are presented. Firstly, the diagrammatic approach should be chosen based on the type of data needed. Secondly, appropriate instructions will depend on the approach chosen. And thirdly, the final results should present examples of original or recreated diagrams. This review also highlighted the need for a standardized terminology of the method and a supporting theoretical framework.

## Background

Diagrams are graphic representations used to explain the relationships and connections between the parts it illustrates. There are many subcategories of the broader term 'diagram', which are distinguished by the elements they incorporate or their overall topic. Two dominant subcategories include 'concept maps' and 'mind maps'[1]. Diagrams are typically brought into the research process in later stages of data analysis or when summarizing and presenting final results. It is commonplace to see a diagram illustrating how concepts or themes relate to each other or to explain how the research data relates to an underlying theory. These diagrams can be developed through the researchers' inductive reasoning of the data collected or may be assisted by computer software[2].

The use of diagrams in earlier stages of the research process (i.e. to collect data) is a relatively new method and is not a common data collection approach at present. However, their use is developing in multiple disciplines, including healthcare research. Diagrams have been used to collect data from research subjects by asking them to either draw a diagram themselves or modify a prototypic diagram supplied by the researcher. The use of diagrams in data collection has been viewed favorably in helping to gather rich data on healthcare topics. These research topics are widely varied and include collecting information to improve patient safety with medication[3], understanding neighborhood characteristics related to mental well-being[4], mapping out healthcare networks[5], evaluating patient educational programs[6,7], understanding how different populations view microbial illnesses[8], diagramming as part of nursing education that is evidence-based[9] and involves critical thinking[10,11], to engage youth in healthcare consultations[12], and to gain insights on physician professional growth[13] and their accountability relationships[14].

Despite the increasing use of diagrams in data collection, there lacks a strong "supportive structure" (pg. 343) for researchers choosing this method[15]. The use of diagrams in data collection has developed independently in multiple disciplines under a number of different names, making knowledge transfer regarding this technique difficult. For example, little has been published on process mapping outside of the organizational literature until fairly recently[5,16,17]. This has limited the exchange of best practices between disciplines. Researchers are often starting from scratch when designing their diagramming data collection approaches and their analysis of the unique data collected[15].

By conducting a multidisciplinary systematic review, as defined in the PRISMA statement[18], we hope to consolidate lessons learned and offer recommendations for researchers in healthcare and other disciplines about how diagrams may be incorporated into their data collection process. The questions that guided our search for relevant studies were:

(1) What drives the selection of a diagramming approach for data collection?

(2) What are the different approaches to diagramming for data collection?

(3) What are the different approaches to analyzing data collected with diagramming?

(4) What are the benefits and challenges of using diagramming for data collection?

## Methods

Diagramming techniques used for data collection in the research process is a challenging area to review, given the variable terminology across, and even within, fields. A preliminary survey of the literature helped identify some key terminology used in different disciplines (e.g. "graphic elicitation" or "participatory diagramming"). The terms used in the titles and abstracts of the preliminary articles identified, as well as the keywords used to index them in databases, formed the basis of our multidisciplinary search strategy. We combined these specific terms with general 'diagram' terms and with general 'data collection' and/or 'analysis' terms.

In December 2009, we electronically searched 13 indexed sources, including traditional health care related indexes and non-health focused indexes (EMBASE; HealthSTAR; Medline; Cumulative Index to Nursing and Allied Health Literature; GEOBASE; InfoTrac Environmental Issues & Policy eCollection; ProQUEST Dissertations; Design and Applied Arts Index; Education Resources Information Center; International Bibliography of the Social Sciences; PsychINFO; Public Affairs Information Services; and Social Science Citation Index). To ensure that all appropriate references were identified and to limit publication bias, non-indexed sources were also searched via general search tools (i.e. Google Scholar and Google Books) to uncover any additional publications. To supplement the search, 35 experts, identified by our searches, were contacted and asked to identify additional relevant articles and grey literature.

Reference Manager 11 was used to support the review. Following the removal of duplicates, articles were screened based on their title and abstract. The full-text was then screened for articles not excluded based on their title/abstract. Articles were excluded if they were not written in English, did not use diagramming techniques in the data collection process (i.e. research subjects did not create or edit diagrams) or were not evaluative or reflective about the data collection process and/or analysis of data collected from diagramming methods. No publication date or publication type restrictions were imposed; research studies, theoretical articles, method articles and opinion pieces were included if they met the above criteria.

The screening was undertaken by two authors (MJU, PT). Double screening was done at regular intervals to ensure inter-rater reliability. Further, the two researchers met weekly during the screening and data extraction phases to discuss the nuances of the articles and to resolve differences by deliberation until consensus was reached.

## Results

**Figure 1:**
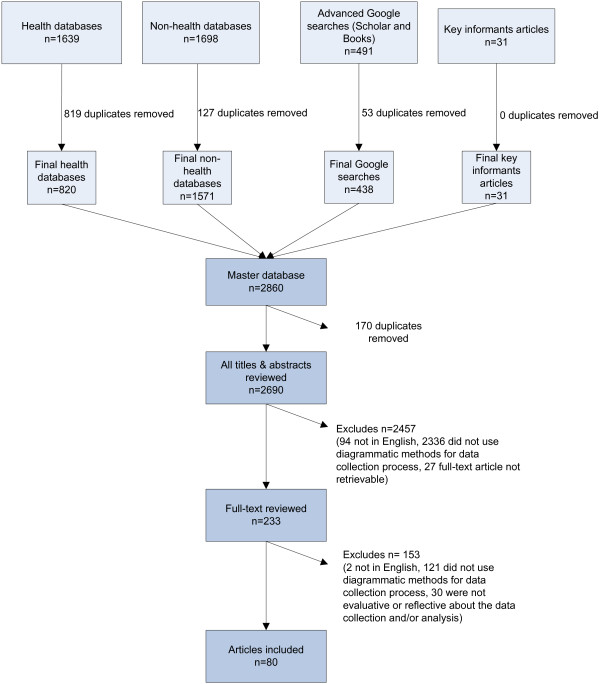
**Flow of articles through the systematic review**.

A total of 2690 references were identified, after the removal of duplicates. Given our search had no publication date restrictions and included dissertations, full-text articles were sometimes difficult to retrieve. Authors were contacted when the article could not be found online or through the University of Toronto's library system. While 4 articles were retrieved in this manner, 27 full-text articles still could not be found and were ultimately excluded. In total, 233 full-text articles were screened and a total of 80 articles were included in the study's review. Figure 1 presents a flow diagram of our search and screening. Data was extracted on the general characteristics of the articles and the four objectives detailed earlier (see Table 1).

**Table 1:** Characteristics of reviewed studies using diagramming data collection approaches

General characteristics of reviewed articles	• Range between 1986 and 2010, with increasing popularity: substantial increase after 2006 • Wide range of disciplines use diagramming data collection approaches: used in the education field most commonly • Number of research subjects varied: average was 36 with a range of 2 to 243 research subjects • Wide range of research participant characteristics: included students, professionals, administrators and laypersons
**Reasons for choosing diagrams for data collection**	• Requirements and challenges of the research topic: e.g., to capture cognitive structure, changes over time and/or differences between groups; to overcome linguistic, cultural, social or intuitional barriers; to collect data on highly complex subject matter • Unique dataset: diagrams seen as a reflective tool, providing holistic coverage through uncensored and unique data

**Approaches for instruction and creation of diagrams**	• Instruction given to research subjects important in shaping end product: ranged from basic requests to create a diagram to specific instructions on what elements to include and practice sessions with feedback • Degree of freedom in diagram creation: data collected by original diagram creation in groups or individually, or through editing a presented diagram or through researcher creation with real-time input • Number of diagrams created or edited: multiple diagrams can be used to track changes over time • Use of other data collection methods: other methods for collecting data were commonly used alongside of diagramming approaches

**Approaches to analysis of diagrams created in data collection process**	• Highly structured diagrams were conducive for quantitative analysis: e.g., counting of elements and/or scoring based on weights assigned to elements of the diagram • Less structured diagrams were conducive for qualitative analysis: e.g., thematic and content analysis • Additional data analysis: diagrams can guide additional data analysis of data collected with other means, provide validation and/or visual representation to illustrate conclusions

**Benefits and challenges of using diagrams to collect data**	• Complementary to other data collection techniques • Helps research subjects to focus and reflect on topic(s) • Benefits/challenges dependent on the application and type of diagram used

### General characteristics

Of the 80 articles included in our review, 53 were published studies[1,4-15,19-58], 19 were dissertations[59-77], 2 were books[78,79] and 6 represented grey literature[3,80-84], including unpublished working papers submitted by key experts and reports available on the internet. These articles were published between 1986 and 2010, with the majority published after 2000 and a substantial increase after 2006. This suggests that interest in these techniques has been increasing in recent years.

The most common discipline, determined by the lead author's affiliation and/or publication title, was from the education field. Other disciplines included healthcare, engineering, environmental science, geography, industrial design, psychology, and social science. The majority of articles clearly specified the study sample size, which averaged 36 research subjects, with a range of 2 to 243. Diagramming methods were used with a wide variety of research subjects, including students (elementary to graduate school), farmers, nurses, physicians, engineers, administrators and graphic designers.

### What drives the selection of a diagramming approach for data collection?

The majority of articles specified at least one explicit reason why a form of diagramming was selected for data collection. These reasons fall into two broad categories: requirements or challenges of the research topic and the unique dataset that results from using diagrams.

The specific research topic examined was the most common reason for researchers choosing a diagramming technique for data collection. For some research topics, past studies have validated diagramming data collection techniques as a useful way to collect data. For example, research has established the usefulness of diagrams in collecting data about research subjects' knowledge or cognitive structures[19-22,59,80]. Diagrams in data collection have also been validated as a means of measuring changes over time[6,10,20,22-24,60,85] and differences between participant groups[20,25,61,81]. Diagramming methods were also sought out when research topics were not conducive to the more common qualitative data collection methods, such as interviews alone. These reasons include a research topic that deals with a population with linguistic, cultural, social or intuitional barriers the researcher wants to overcome[12,14,15,26-29,79] or with highly complex subject matter[12,14,25,30-32,85]. Examples of highly complex subject matters include the abstract nature of the research topic of 'pedagogical constructs'[25] and the multifaceted and diverse nature of 'clinical accountability relationships'[14].

Secondly, researchers sought out diagramming data collection methods because of the benefits previous studies found regarding the quality and uniqueness of the collected dataset. When research subjects drew diagrams without prompts, previous studies concluded that it minimized the influence of the researcher on the participant and their responses[1,33-35,61,62,81,82]. Studies have also found that diagramming is a reflective tool for the research subjects[28,29,36,63]. Since diagrams can represent both concrete and theoretical notions[37], diagramming offers a more holistic coverage of the topic[29,38,61], with more uncensored and unique data gathered[1,24,28,35,39,40,58] than more traditional qualitative data collection methods.

### What are the different approaches to diagramming for data collection?

A range of applications were identified, which varied widely based on the degree of instruction, degree of freedom in diagram creation, the number of diagrams created or edited and the use of diagram in conjunction with other data collection methods.

Half of the studies did not report the details of the instructions provided to the research subjects, except for describing the basic request to create a diagram. One study explicitly observed that specific instructions are needed to ensure the research participants create a diagram and not another form of written material[32], such as a drawing or table. Simple or short instructions were often given to research subjects when the diagram sought by the researchers did not have to conform to a rigid structure, such as life-cycle[13] and professional practice diagrams[32].

When the researcher sought highly structured diagrams, the degree of instruction provided to the research subjects ranged from the preferred method of giving specific and detailed instructions on what elements should be included (e.g. hierarchies, arrows) to showing an example of the type of diagram the researcher would like the participant to create. For example, in comparison to other diagramming techniques, concept maps have a fairly rigid definition and a very specific set of elements that the end diagram should contain. Such diagrams may require more detailed instructions. One study had associate nursing degree students draw their own concept maps after a 20-minute introductory tutorial, presentation of a sample diagram, discussion and question period, and instructions listing all elements to be included (e.g. arrangement of items, hierarchal order, linking concepts with arrow, labeling propositions, identifying cross-links/relationships)[9]. In some instances, research subjects were given the opportunity to practice the diagramming method and receive corrective feedback prior to data collection[19,23,41,64].

It was most common for research subjects to create an original diagram on their own, in groups or a combination of both. Alternatively, some studies had the participant edit either a designated diagram provided by the researcher[3,14,15,42] or a researcher-created diagram generated by the researcher during the interview with participant input[4,5,7,43]. A few studies also chose the middle ground between original and prepared diagrams. For example, some provided a central concept or word to create the diagram around[14,44], or included some words or shapes to fill in on a prepared diagram[45] and others gave research subjects a list of words to use in the creation of their diagram[65,80].

Half of the studies used diagrams at multiple times within the data collection process as a means of comparison. A subset used a pre-/post- (or time series) approach to data collection, allowing researchers to track changes before, after, and sometimes during an intervention. This was found primarily within the discipline of education. For example, Rios asked a sample of teachers to create concept maps at multiple time intervals in order to identify their conceptual models and examine the impact of student interactions on the teachers' subject matter structure or vice versa[66].

Some researchers explicitly expressed the idea that diagrams alone would not capture complete perspectives from the research subjects[57,77], suggesting that diagrams should be used in combination with other data collection methods. The majority of the studies did use diagramming data collection techniques in addition to other methods. In some cases the additional data collection methods were used explicitly in conjunction with the diagramming techniques, such as creating the diagram within interviews or discussion of the diagrams in later focus groups or interviews[8,23,39,59,60,67].

### What are the different approaches to analyzing data collected with diagramming?

The majority of the articles reported details about the analysis of diagram data. The use of only quantitative or qualitative analysis, or a mixture of both types of analysis was fairly equally distributed among the articles. Within each of these three categories of analysis, there were a variety of different techniques used that are briefly outlined below.

The majority of studies comparing diagrams across time or across research subjects chose either quantitative techniques only or a mix of qualitative and quantitative techniques for their analysis. Quantitative analysis techniques included counting (e.g. number of concepts identified, number of links between concepts, number of examples given, levels in hierarchy) and scoring. The two most common scoring methods were structural scoring and relational scoring[22]. Structural scoring refers to when weights are assigned to hierarchical structures, links between concepts and other elements. For example, a link between two concepts may be 10 points, an example 1 point and invalid examples and cross links 0 points[40]. Relational scoring reflects the quality or importance of each concept or link as determined by the researcher, by comparison to a similar diagram created by an expert or by other research subjects[36,38,60,62,68]. Some studies using quantitative analysis showed diagrams to illustrate how the final counting and scoring of a diagram was completed in the presentation of final study results[35,40,41,46-49,54,62]. For example, Kesby had focus groups in Zimbabwe create scored diagrams with local materials of rocks, string and bottle caps[54]. These diagrams were photographed and also reproduced on computer for legibility. Including an original diagram in the presentation of results also helps to orient the reader to the type of diagram that was used.

Studies which had diagrams completed or edited in the presence of the researcher, included additional data collection methods, and/or studies using less structured diagrams to collect data were likely to use either qualitative techniques only or a mix of qualitative and quantitative techniques for their analysis. For example, Haidet et al. gave medical school research subjects less structure and encouraged creativity in their diagramming exercise within interviews[13]. The diagram was used as a prompt to stimulate discussion, which was then analyzed through the interview transcripts. The diagram itself was then displayed in the final results to visually summarize the verbal exchange. Qualitative analysis included thematic and content analysis of the diagrams and additional data sources, such as transcripts, to identify prominent topics, themes and patterns in the diagrams[13,25,26,34,63,66,69-71].

In some studies that used mixed-methods, the collected diagrams were the primary source of data and guided the analysis of additional data sources, for example, by providing the core themes for transcript analysis. In other cases the additional data sources played the dominant role in analysis and the diagrams were used almost as verification or visual representation to illustrate conclusions[72].

### What are the benefits and challenges of using diagramming for data collection?

Some of the benefits to using diagrams for data collection have already been discussed in the section on why researchers chose diagramming data collection approaches. In addition to these, diagramming approaches that were seen as complementary to other data collection approaches were commonly used in interviews and focus groups[60,73]. They were found to help focus discussions on particular themes[32] and enabled research subjects to more easily reflect on a topic or their beliefs by helping them to express thoughts in a more structured and organized manner[50,51]. The use of diagrams was also seen to increase recall[52] and self-reflectiveness[53,54]. In 1992, Powell found that interviews which made use of diagramming approaches were more introspective and tended to be more theoretical and philosophical than those that did not use diagramming methods[25].

Over half of the articles discussed at least one challenge of using a diagramming method for data collection. Interestingly, these challenges were often contradicted by other articles. All studies completed their data collection with diagrams, with some reporting that the diagramming allowed research subjects to overcome challenges of verbal communication[12,14,15,26-29,75,79]. However, many studies found that at least some of the research subjects expressed difficulty or discomfort with the diagramming task[1,9,10,14,28,35,55,56,58,62,74,76,83]. Some identified the ease and speed of data collection as benefits of using diagramming approaches[24,31,54], while others saw it as being time-intensive, particularly for analysis[6,11,26,62]. Related to the visual organization and structure of knowledge that the diagrams presented, an advantage to using diagramming approaches for data collection is their ability to obtain unique and unsolicited data[7,14,15,26,29,33,42,43,58]. In contrast, there were also concerns regarding the data it did not collect, such as non-verbal communication, that require the discretion and experience of researchers to identify and interpret[15,24,84]. These contradictions illustrate that the benefits and challenges to using diagramming approaches for data collection depend on the application and type of diagram used in each research study.

## Discussion

This systematic review represents the first overview of diagrams being used as a data collection approach in multiple disciplines. In 2006, Nesbit & Adesope[86] conducted a widely cited meta-analysis looking at peer-reviewed articles focusing on learning with concept and knowledge maps and found that the interest in using diagrams appeared to be on the rise[86]. While our systematic review concurs that interest is growing, it differs from their meta-analysis in two ways. Firstly our definition of a diagram is much broader, encompassing a variety of diagrams that extend beyond concept and knowledge maps. As well as the structured diagrams that Nesbit & Adescope focused on, our review also includes less structured diagrams, such as the life-cycle and professional practice discussed in our results section. Secondly, our focus is solely on diagrams being used as a data collection approach, whereas their meta-analysis included diagrams used as analysis techniques as well.

Given our broad definition of a diagram, we have reviewed approaches for collecting data through a wide spectrum of diagrams, from highly structured concept maps to less defined diagrams. We have provided an overview of the instruction options for research subjects, the creation and analysis of diagramming as a data collection approach, as well as highlighted some of the benefits and challenges. While there is variation regarding the guidance in instructions and approaches to the construction of different diagrams, use of diagrams as a data collection tool as a whole is clearly increasing in healthcare and in other disciplines. This systematic review is the first step in consolidating this information to assist in the refinement of this approach. For those considering using diagramming as a data collection approach, we offer three recommendations. Firstly, the diagrammatic approach should be chosen based on the type of data needed to answer the research question(s). Secondly, based on the diagrammatic approach chosen, it is important to select the appropriate instructions needed. Finally, presentation of final results should include examples of the original or recreated diagrams.

### Choice of Diagramming Approach

The most important considerations for choosing the diagramming approach is the type of data needed to answer the research question (e.g., examining change over time, exploring people's experiences or views) and the type of analysis preferred by the researcher. For example, highly structured diagrams allowed for valid quantitative analysis, such as counting and ranking, which could be compared across research subjects. It should be noted that there is some controversy whether items should even be counted and that both an over- or under-reliance may be dangerous to the final research conclusions[28]. In comparison, other approaches that used a less structured diagrams relied heavily on qualitative analysis.

### Instruction and Creation

The appropriateness of different approaches to instructions to guide diagram creation is an important consideration in ensuring the validity of data. It is clear from our review that the initial instructions given to research subjects varied in structure but had a great impact on the resulting diagrams and their potential for different analysis techniques. If researchers require highly structured diagrams it may be useful to give research subjects more detailed instructions and the opportunity for practice and feedback[30,64].

### Presentation of results

The last recommendation is that studies using diagramming data collection approaches should include visual presentations of the findings in their results sections. The use of diagrams often results in the collection of unique and unsolicited data through a visual component, which can then be displayed along with the final analysis. Just as diagrams can provide data not easily obtained through verbal data collection techniques, visual presentation of the collected diagrams may provide insights not as easily grasped through verbal communication of study results. While providing a scanned image of the original diagram can be difficult at times, it is also possible to present a photograph of the original diagram or a computer-generated recreation of the diagram. This is especially important given the variation in terminology between disciplines, as it relays to the reader the type and structure of diagrams created or used.

In addition to the recommendations for researchers considering the use of diagramming data collection approaches, this multidisciplinary review also identifies areas where future research is needed. This review required a substantial amount of preliminary work to understand the terminology used to describe diagrams and this data collection approach across different disciplines and fields. The intent was to devise a sensitive search, so as to cast a wide net in order to capture articles in a range of disciplines where the terminology is not standardized. Cole et al. illustrate this issue by identifying over a dozen different terms that are used to describe concept maps[56]. Thus far, development of terminology has focused on the end result, i.e. what type of diagram is created based on the elements it contains, rather than focusing on the actual data collection approach itself. This has created different data collection approaches that use diagrams separately, isolating research done across disciplines and even within disciplines. Therefore, it is our recommendation that efforts are directed towards standardizing the terminology for this data collection method. This would allow researchers to maintain the work they have done regarding specific types of diagrams, whilst providing an umbrella term to help with the sharing of best practices. Future research should also be directed at identification of the underpinning theory of the method as the review demonstrated a gap in this area[15,32,35]. Such a theory may help to further inform researchers regarding the appropriate use and applications for diagramming data collection approaches.

A limitation of our review is that the database search strategy did not capture general articles on visual data collection methods, which may include specific information on diagramming data collection approaches. However the non-indexed searches and articles identified by experts did pick up some of these articles. While efforts were made to contact authors to retrieve articles from our search, twenty-seven full-text articles were irretrievable. Ten of these were dissertations and five were books. This may have contributed to an incomplete representation of what the literature has to offer about diagramming data collection approaches.

## Conclusion

There has been a growing interest in the use of diagrams for data collection in the research process over recent years, as shown by the increase in publications and the wide range of approaches developed for diagramming data collection and diagram data analysis. As noted earlier, diagrams have been used to collect rich data on a variety of healthcare topics and it is expected that the use of this method will continue to grow. The results of this multidisciplinary systematic review provide an overview of the application of diagrams in research data collection and the methods for analyzing the unique datasets elicited. Recommendations are presented to assist researchers considering the use of diagrams in their data collection process. This review also highlighted the need for a standardized terminology of the method and a supporting theoretical framework.

## Competing interests

The authors declare that they have no competing interests.

## Authors' contributions

MJU, MJD, HEDB participated in the conception and design of the study. Data collection, abstraction and analysis was carried out by MJU and PT. MJU and PT prepared the original draft of the manuscript, and all authors reviewed and critically revised the original and subsequent manuscript drafts and approved the final manuscript.

## Pre-publication history

The pre-publication history for this paper can be accessed here:

http://www.biomedcentral.com/1471-2288/11/11/prepub
